# Identification of functional tRNA-derived fragments in senescence-accelerated mouse prone 8 brain

**DOI:** 10.18632/aging.102471

**Published:** 2019-11-20

**Authors:** Shuai Zhang, Hejian Li, Ling Zheng, Hong Li, Chengqiang Feng, Wensheng Zhang

**Affiliations:** 1 Zhuhai Branch of State Key Laboratory of Earth Surface Processes and Resource Ecology, Beijing Normal University, Zhuhai, Guangdong 519087, China; 2 Engineering Research Center of Natural Medicine, Ministry of Education, Faculty of Geographical Science, Beijing Normal University, Beijing 100875, China; 3 Beijing Key Laboratory of Traditional Chinese Medicine Protection and Utilization, Faculty of Geographical Science, Beijing Normal University, Beijing 100875, China; 4 National and Local United Engineering Research Center for Panax Notoginseng Resources Protection and Utilization Technology, Kunming, Yunnan 650000, China

**Keywords:** tRNA-derived fragments, brain aging, Alzheimer’s disease, Parkinson's disease, senescence-accelerated mouse prone 8

## Abstract

Transfer RNA-derived fragments (tRFs) are known to contribute to multiple illnesses, including cancers, viral infections, and age-related neurodegeneration. In this study, we used senescence-accelerated mouse prone 8 (SAMP8) as a model of neurodegenerative disorders such as Alzheimer’s disease and Parkinson’s disease, and a control, the senescence-accelerated mouse resistant 1 (SAMR1) model, to comprehensively explore differences in tRF expression between them. We discovered 570 tRF transcripts among which eight were differentially expressed. We then obtained 110 potential target genes in a miRNA-like pattern. Gene Ontology (GO) and Kyoto Encyclopedia of Genes and Genomes (KEGG) annotation suggest that these target genes participate in a variety of brain functions; *e.g.*, synapse formation (GO: 0045202) and the synaptic vesicle cycle pathway. We further assessed in detail those tRFs whose miRNA-like pattern was most likely to promote the progression of either Alzheimer’s or Parkinson’s disease, such as AS-tDR-011775 acting on *Mobp* and *Park2*. Our findings suggest the eight dysregulated tRFs we uncovered here may be beneficially exploited as potential diagnostic biomarkers and/or therapeutic targets to treat age-related brain diseases.

## INTRODUCTION

Changes occur in all parts of a person’s body, including the brain, during aging. The brain naturally shrinks in volume, and an increased size of the brain sulci with age is observed [1]. These changes have significant impacts on learning and other complex mental activities. Thus, brain aging and neurodegeneration appear to go hand in hand, especially in Alzheimer’s disease (AD) and Parkinson’s disease (PD) [2]. Some countries, such as China, Japan, and Italy, have become aging societies that have considered the problem of aiding the aging brain to prevent related diseases as a public concern. Many theories such as Aβ deposition and tau phosphorylation in AD [3] and alpha-synuclein aggregation in PD [4] have attempted to explain the causes of these pathologies. In spite of the knowledge gained concerning the etiology of AD and PD in the last several decades, few treatments are available to prevent them.

In gene expression regulation, post-transcriptional regulatory molecules interact with specific non-coding RNAs (ncRNAs) including miRNAs, piRNAs, snoRNAs, lncRNAs, and circRNAs. ncRNAs participate in complex mechanisms of brain aging and related diseases; for example, circulatory miR-34a is an accessible biomarker for age-dependent changes in the brain [5], and piRNAs may be involved in age-dependent histone control of complex networks of memory-related genes [6]. Small ncRNAs derived from tRNAs are called tRNA-derived fragments (tRFs), with lengths ranging from 14 to 36 nucleotides (nt) [7, 8]. They can be classified into tRF-5, tRF-3, tRF-1, i-tRF, and tiRNA (tiRNA-3 and tiRNA-5) [9, 10]. Many studies have shown that tRFs contribute to diverse illnesses, such as cancer and viral infectious disease [11–13], mammalian brain aging [14], and other neurodegenerative processes [15, 16]. However, the tRF functions in common brain-aging diseases such as AD and PD are poorly understood.

The senescence-accelerated mouse prone 8 (SAMP8) model has approximately half the normal lifespan of a wild-type (WT) rodent and displays early-onset senility characterized by memory and learning ability deterioration [17]. SAMP8 is thus a good model to study brain aging and related disorders, such as AD and PD [18, 19]. On the other hand, the senescence-accelerated mouse resistant 1 (SAMR1) strain undergoes normal aging and is often used as a control [20]. In our previous studies, we analyzed changes in lncRNA, circRNA, miRNA, and DNA methylation in the brain of SAMP8 as well as the related mechanisms regulating gene expression [21–23]. In this study, we used deep RNA sequencing to test whether SAMP8 brains exhibit changes in tRF expression relative to controls. We also determined how such changes affect brain aging and the pathology patterns of AD and PD. Our research is the first to provide systematic insights into the profiling of the tRF transcriptome in the brain aging model SAMP8. These tRFs may be potential therapeutic targets and diagnostic markers for brain aging-associated illnesses, primarily AD and PD.

## RESULTS

### SAMP8 mice exhibit a decline of learning and memory at the 7-month stage

Morris water maze (MWM) test was performed to evaluate the learning and memory deficits in 7-month-old SAMP8 mice. The result for the hidden platform test is shown in Supplementary Figure 1A. SAMP8 mice took a longer time to find the platform than SAMR1 mice (*p* < 0.05). The spatial probe test was then performed. Supplementary Figure 1B clearly illustrates that the SAMR1 mice searched for the destination location purposefully, whereas the SAMP8 mice swam aimlessly in the pool. The number of crossings and the time percentage in the target quadrant were significantly lower for the SAMP8 group than for the SAMR1 group (*p* < 0.05, Supplementary Figure 1C and 1D). With regard to swimming speed, no difference was observed between the two groups. (*p* > 0.05, Supplementary Figure 1E). This result suggests a lack of motor and visual dysfunction in the SAMP8 mice. On the other hand, the 7-month-old SAMP8 mice presented impaired memory and poor learning skills. These findings were consistent with the clinical neurophysiology of the aging brain and related neurodegeneration clinical symptoms.

### Altered expression profiles of tRFs in the SAMP8 mouse brain

A total of 69,772,438 raw reads (34,909,558 for the SAMP8 mice and 34,862,880 for the SAMR1 mice) were generated. After the 5ʹ- and 3ʹ-adaptors were trimmed, low-quality reads were removed, and ≤16 bp reads were filtered. A total of 68,118,335 clean reads (33,886,463 for SAMP8 mice and 34,231,872 for SAMR1 mice) were found in the two groups. Most clean reads were 22, 21, 23, and 45 nt in length for both groups (Supplementary Figure 2A and 2B). Then, the high-quality clean data were mapped to the mouse mature-tRNA and pre-tRNA sequences from GtRNAdb by NovoAlign software (v2.07.11). In accordance with the comparison results, 570 tRFs were detected. These tRFs were used for subsequent analyses.

**Figure 1:**
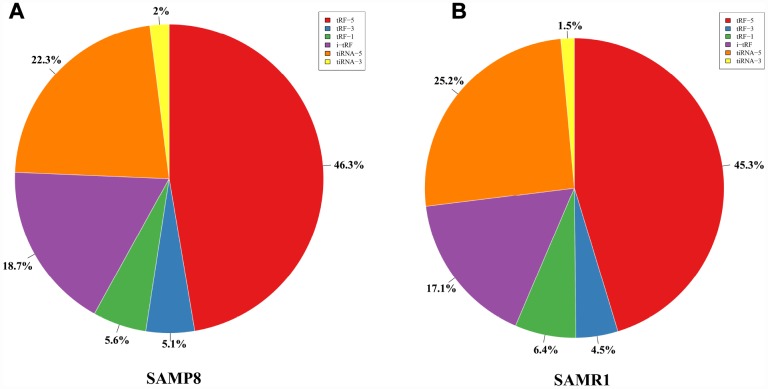
**Proportions of tRF-5, tiRNA, i-tRF, tRF-3, and tRF-1 in the two groups.** (**A**) Proportions in SAMP8 mice. (**B**) Proportions in SAMR1 mice.

**Figure 2:**
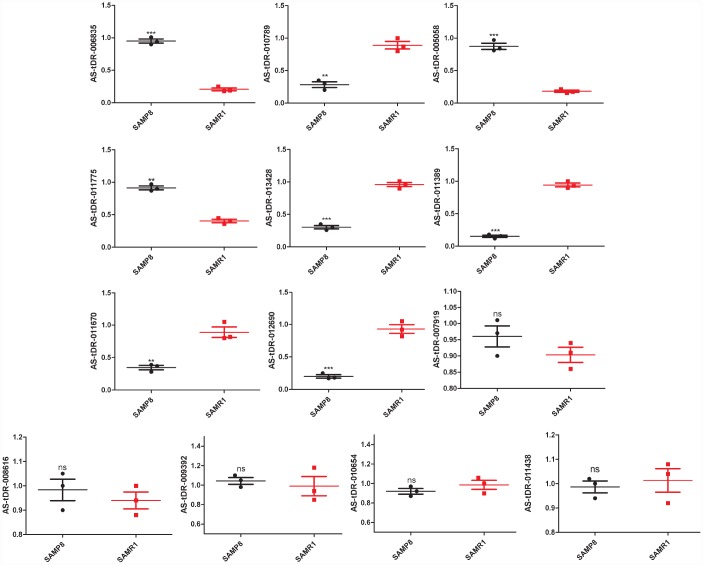
**Validation of tRFs expression by quantitative polymerase chain reaction (qPCR).** The U6 gene was used as a housekeeping internal control. The relative expression of each tRF was represented as mean ± SEM [n = 3, three mice per group (biological replicates), three times per mouse (technical replicates)]. *p < 0.05, **p < 0.01, ***p < 0.001, ns means nonsignificant.

**Figure 3:**
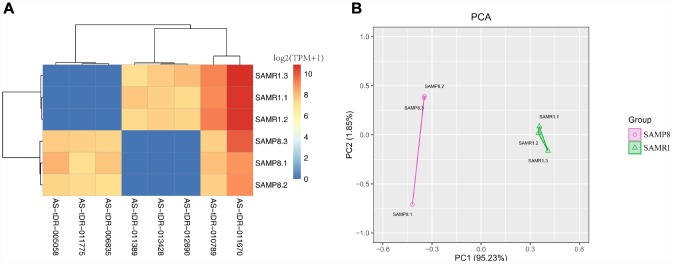
**Cluster analysis and principal component analysis of differentially expressed tRFs in the SAMP8 vs SAMR1 mice.** (**A**) Cluster analysis. (**B**) Principal component analysis.

We used transcripts per million (TPM) to estimate the expression of the tRF transcripts. The levels of each subtype showed a similar proportion between the two groups. The percentages were approximately 45% tRF-5, 26% tiRNA (2% tiRNA-3 and 24% tiRNA-5), 19% i-tRF, 5% tRF-3, and 5% tRF-1 (Figure 1A and 1B). As a result, 13 differentially expressed tRFs were identified (*p* < 0.01 and fold changes ≥2). To validate the changes detected by RNA-seq, all 13 tRFs were selected, and their expression was further examined by quantitative polymerase chain reaction (qPCR). As shown in Figure 2, eight of the 13 transcripts whose levels were measured showed differential expression in SAMP8 and SAMR1 brains (*p* < 0.01, Supplementary Table 1). This result was inconsistent with the RNA-seq data possibly because of the biological differences between samples. Then, principal component analysis and cluster analysis were performed for the eight differentially expressed tRFs (Figure 3A and 3B). In the SAMP8 group, three samples were clustered together. The same situation occurred in the SAMR1 group.

### Functional enrichment analysis revealing the close correlation between tRFs and brain function

Fu *et* *al.* discussed that tRFs participate in translation regulation and gene silencing [24]. Among them, an important pattern is the miRNA-like behavior [25, 26]. On the basis of this concept, we pioneered the identification of tRF-mRNA pairs in the SAMP8 brain through mRNA-seq [21] and tRFs-seq data. The results are presented in Supplementary Table 2. One-hundred ten potential target genes were identified.

Gene Ontology (GO) survey was performed on the abovementioned target genes. As a result, 168 GO terms were enriched (adjusted *p* value < 0.01, Supplementary Table 3). Importantly, several brain function-associated terms were detected, including postsynaptic membrane (GO:0045211), postsynaptic density (GO: 0014069), and synapse (GO:0045202). By contrast, results from analyses of the Kyoto Encyclopedia of Genes and Genomes (KEGG) revealed 14 enriched pathways (*p* value < 0.01, Supplementary Table 4). Brain function-associated pathways were also detected, including synaptic vesicle cycle, axon guidance, and dopaminergic synapse. Overall, these protein-coding genes may be regulated by tRFs by using a miRNA-like pattern in the SAMP8 mouse brain.

### Functional specificities of tRFs in AD and PD

To obtain an improved understanding of the relationship between tRFs and brain aging and related diseases, especially AD and PD, we set three restrictions. For the first factor, the tRFs and their target genes must be expressed differently between the SAMP8 and SAMR1 mice. For the second factor, the trend of expression of tRF and its target gene should be the opposite in the brain. These trends include two cases. One case corresponds to the tRF (upregulated in SAMP8 mice)-mRNA (downregulated in SAMP8 mice), and the other case refers to the tRF (downregulated in SAMP8 mice)-mRNA (upregulated in SAMP8 mice). For the last requirement, the selected pairs (tRF-mRNA) should promote pathological processes in AD and PD. If a pair meets the above criteria, it can be selected. For example, Camk2n1, an endogenous CaMKII inhibitor protein, showed a direct effect on synaptic CaMKII-NMDAR binding and played an important role in LTP regulation [27]. We also found that the *Camk2n1* expression in the SAMP8 mice brain was higher than that in SAMR1 mice. AS-tDR-011389 was present in low levels in the SAMP8 mouse brain and targeted *Camk2n1*. *Rpsa*, a gene that facilitates the production and internalization of neurotoxic Aβ peptide [28], was targeted by AS-tDR-013428. AS-tDR-011775 acted on *Mobp*, which may contribute to determining the morphology of axons in neurons [29]. Park2 contributes to Parkinson’s disease [30]. *Park2* was targeted by AS-tDR-011775. P2Y1 receptor protein is encoded by the *P2ry1* gene. A study confirmed that the P2Y1 receptor contributed to astroglial network dysfunction by purinergic signaling in AD [31]. *P2ry1* was regulated by AS-tDR-011389. AS-tDR-005058 acted on *Erc1.* A previous study showed that neurotransmitter release can be regulated through the Rab6ip2/ERC1/CAST2/ELKS and presynaptic active zone protein interaction [32]. The specific details are listed in Table 1. We predicted that these tRFs most likely participate in the occurrence and development of AD and PD.

**Table 1:** tRFs are most likely involved in the pathogenesis of AD and PD through a miRNA-like pattern.

**tRFs**	**SAMP8_TPM**	**SAMR1_TPM**	***P* value**	**Potential target gene**
AS-tDR-005058	224.88	0.00	0.004333786	Erc1 (SAMP8↓)
AS-tDR-011775	162.75	0.00	5.6518E-05	Mobp, Park2 (SAMP8↓)
AS-tDR-011389	0.00	176.24	0.00103563	P2ry1, Camk2n1 (SAMP8↑)
AS-tDR-013428	0.00	192.05	2.25358E-05	Rpsa (SAMP8↑)

Note: SAMP8↓ means the gene is downregulated in SAMP8 mouse brain; SAMP8↑ means the gene is upregulated in SAMP8 mouse brain

## DISCUSSION

In the year 2009, Cole *et al*. first identified tRFs from cultured HeLa cells [33]. Then, tRFs were detected in other kinds of human cells or tissues [11, 12, 34], as well as in plants [35] and animals [36]. Given their widespread presence, tRFs are hypothesized to play key roles in many physiological and pathological processes, including aging. Getting older underlies cognitive decline and dementia and is the greatest contributor to brain function failure. AD and PD are the two most common types of brain aging-related neurodegenerative disorders, both hard to cure [37]. Recent studies have shifted focus to dysregulated gene regulations in brain aging and related diseases, particularly AD and PD. We have conducted considerable work previously on this aspect, including lncRNA, miRNA, circRNA, and DNA methylation [21–23]. With aging, tRFs undergo dynamic changes in the mammalian brain [14]. Thus, tRFs may be correlated with brain aging and related disease development and progression by regulating the expression of specific genes. The primary goal of this study was to identify tRFs involved in brain aging and related diseases. Collecting human tissue samples is difficult for several reasons. Thus, we worked with an animal model instead. SAMP8 mice are known to exhibit age-related brain degeneration and AD and PD-like pathologies [19, 38]. Our MWM test confirmed that SAMP8 mice exhibited memory impairments and learning deficits at 7-month of age. These symptoms are core clinical features observed among elderly people. Moreover, we used high-throughput sequencing (RNA-seq) to identify potential tRFs with unprecedented detail. Our results warrant further studies on candidate tRFs to test their potential use as novel therapeutic targets or reagents to treat AD and PD.

Karaiskos *et al*. described the age-driven modulation of tRFs in Drosophila but focused specifically on the tRF species containing CCA at the 3′-end [25]. Interestingly, we also discovered several 5ʹ-derived tRFs (94 tRF-5 and 69 tiRNA-5), 232 i-tRFs, and 67 tRF-1 in our present work. In general, tiRNA-5, tiRNA-3, tRF-3, tRF-5, and i-tRF series, which are generated from mature tRNAs, constituted the majority in our samples. Meanwhile, the tRFs generated from primary tRNAs (tRF-1) were in the minority. This phenomenon is consistent with previous reports [36, 39, 40].

However, although i-tRF was the subtype with the most tRF transcripts in number, the levels of the i-tRF transcripts were not the highest. The tRF-5 subtype showed the highest expression. The 5ʹ-derived tRFs (tRF-5 and tiRNA-5, approximately 70%) were the most abundant class of tRFs between the two groups, followed by i-tRF (approximately 18%), with the 3ʹ-derived tRFs (tiRNA-3, tRF-3, and tRF-1, approximately 12%) as the least (Figure 1). tRNAs with different sequences may have the same anticodon and transfer the same amino acid. tRNAs were divided into many groups depending on their anticodon [41]. Our analyses showed that these tRFs are derived primarily from Lys-CTT, Gly-GCC, Gly-CCC, Glu-CTC, Glu-TTC, Val-CAC, Val-AAC, Met-CAT, Ala-TGC, Gln-CTG, His-GTG, Asn-GTT, Leu-TAA, Leu-TAG, Leu-CAA, Leu-CAG, Pro-TGG, Pro-AGG, Asp-GTC, Cys-GCA, Tyr-GTA, Thr-CGT, Arg-ACG, Arg-CCT, and Ala-AGC, and partly from Ala-CGC, Lys-TTT, Ser-GCT, and Pro-CGG. Future experiments should focus on determining whether these tRNAs can affect SAMP8 mouse brain aging through the generation of tRFs.

Normally, differential expression is important to understand the biological differences between different physiological or pathological conditions. In our study, 13 differential tRF transcripts were preliminary found via computational analyses. Then, we used qPCR to test the reliability of the initial results. Finally, eight dysregulated tRFs were detected. This finding demonstrated that our pipeline was relatively high-quality strict in identifying putative tRFs and laid a solid foundation for further exploration and experimentation. Herein, AS-tDR-011775 belongs to the tRF-1 subtype. AS-tDR-006835 and AS-tDR-012690 are tRF-3. AS-tDR-013428 is tRF-5. AS-tDR-005058, AS-tDR-011389, AS-tDR-010789, and AS-tDR-011670 are i-tRF. Interestingly, although 5ʹ-derived tRFs (tRF-5 and tiRNA-5) had the highest expression, other types of tRFs appear to serve important purposes in brain aging. Since tRFs often contribute to disease [24, 42], the dysregulated tRFs we uncovered here might contribute to brain aging and related diseases.

We then investigated the specific GO and KEGG enrichments for tRFs-mRNA correlations. Specifically, GO and KEGG enrichments from these tRF-targeting genes pertained to brain functions, such as synapse formation (GO: 0045202) and synaptic vesicle cycle pathways. Furthermore, we selected the most likely tRF-mRNA pairs involved in the regulation of AD and PD progression. Among these pairs, one tRF can dominate an increased number of genes. AS-tDR-011389 is a good example that controls *P2ry1* and *Camk2n1*. This observation suggests that the miRNA-like mechanism of tRFs in the regulation of gene expression is complex in brain aging. Herein, we reiterate that aside from the miRNA-like pattern, tRFs can mediate post-transcriptional modulation in other ways. For example, tRFs displaced the eIF4G translation initiation factor from mRNAs and participated in post-transcriptional regulation [43]. tRFs also competed for the mRNA binding sites of YBX1 to suppress cancer progression [44]. Therefore, the functions of these eight differentially expressed tRFs may be to delay brain aging and the onset of AD and PD. Future studies are required to undertake the great challenge of elucidating the detailed molecular mechanisms underlying the functions of these tRFs.

In summary, our study investigated tRF profiles in the brains of SAMP8 and SAMR1 mice at 7 months of age and found several dysregulated tRFs whose expression patterns may be exploited as potential diagnostic biomarkers for brain aging and related diseases, especially AD and PD.

## MATERIALS AND METHODS

### Preparation of animals

In this study, we purchased SAMP8 mice (n=5, 3 months of age, male, pathogen and virus free, RRID: MGI: 2160863) and SAMR1 mice (n=15, 3 months of age, male, pathogen and virus free, RRID: MGI: 2160867) from Beijing WTLH Biotechnology Co., Ltd. (License No. SCXK Jing 2011-0012). The mice were maintained in separate cages with standard conditions and allowed food and water freely until 7 months old. No animals died and were excluded. All mice were used in subsequent experiments. Eight animals of each group were randomly selected for the MWM test, numbered 1 to 8. The remaining mice were given isoflurane anesthesia, a nonflammable liquid administered by vaporizing and inhalation. Briefly, we first placed the animal in the induction chamber. Second, we adjusted the flowmeter to 0.8 L/min to 1.5 L/min. Third, we adjusted the isoflurane vaporizer to 3%–5%), euthanized by cervical dislocation, and dissected to obtain their cerebral cortices. The tissues were immediately preserved in liquid nitrogen at −196°C for tRF sequencing and other experiments.

All experiments on the mice complied with the “Guide for the care and use of laboratory animals” [45] and were permitted by the Institutional Animal Care and Use Committee of Beijing Normal University (BNU NO. 2018).

### Behavioral studies

The spatial learning and memory of SAMP8 mice at the 7-month-old stage were evaluated through the MWM as previously described [46]. Briefly, the mice were familiarized with the MWM environment on the day before the program. In the hidden platform experiment (days 1–5), we set a platform in one of the quadrants. The mice were trained twice a day for 5 days. The mice were then allowed to swim for 90 s during each training. The escape latency was recorded through a special software once they touched the platform. However, if a mouse failed to reach the platform within the stipulated time, we helped it find the platform, and the escape latency was regarded as 90 s. In the spatial probe experiment (day 6), we withdrew the platform and allowed the mice to swim freely for 1 min. The time spent in the target quadrant, the number of platform crossing, and the swimming trajectory of each mouse within 1 min were recorded. All experiments were performed simultaneously every day, and the investigator was unaware of the mouse genotypes throughout the trial.

### Library preparation

Six cDNA libraries were constructed; *i.e.*, three for the SAMP8 mice and three for the SAMR1 mice. Agarose gel electrophoresis and a NanoDrop ND-2000 instrument (Thermo Scientific™, USA, #ND-2000) were used to examine the integrity and quantity of each RNA sample, respectively. Total RNA samples were first pretreated as follows to remove some RNA modifications that interfere with small RNA-seq library construction: 3ʹ-aminoacyl deacylation to 3ʹ-OH for 3ʹ adaptor ligation, 3ʹ-cP removal to 3ʹ-OH for 3ʹ adaptor ligation, 5ʹ-OH phosphorylation to 5ʹ-P for 5ʹ-adaptor ligation, m1A and m3C demethylation for efficient reverse transcription. The following steps were conducted to prepare a gene library: 1) 3ʹ-adapter ligation, 2) 5ʹ-adapter ligation, 3) cDNA synthesis, 4) PCR amplification, and 5) size selection of ~135–160 bp PCR amplified fragments (corresponding to ~15–40 nt small RNAs). The Agilent bioanalyzer 2100 system (Agilent, USA, #G2939BA) was used to assess library quality. Finally, the libraries were pooled in equal amounts depending on the quantification results.

### Sequencing

The libraries were denatured and diluted to a loading volume of 1.3 mL and loading concentration of 1.8 pM with 0.1 M NaOH. The diluted libraries were then loaded onto a reagent cartridge and forwarded to a sequencing run on the Illumina NextSeq 500 system (RRID: SCR_014983) by using a NextSeq 500/550 V2.5 kit (Illumina, USA, #FC-404) in accordance with manufacturer’s instructions. Sequencing was carried out in 50 cycles.

### Quality control and mapping summary

Raw data files in FASTQ format were generated through Illumina NextSeq 500. The sequencing quality was shown by quality score, represented by Q, which is the −10×log10 transformed probability of the base calling being incorrect. Q30 means incorrect probability of 0.001. If the number is larger than 30, the incorrect probability is less than 0.001; *i.e*., >99.9% correct. In general, when most of the quality scores are above 30, the sequence is of high quality.

After Illumina quality control, the sequencing reads were 5ʹ,3ʹ-adaptor trimmed, filtered for ≥16 nt by the Cutadapt software [47], and aligned to mature-tRNA and pre-tRNA sequences from GtRNAdb [48] by using the NovoAlign software (v2.07.11) [49].

### Expression analysis

tRF expression levels were measured and normalized as read counts per million of total aligned tRF reads. tRFs with fold changes ≥ 2 and *p* < 0.01 were selected as the significantly differentially expressed tRFs between SAMP8 and SAMR1.

### Quantitative real-time PCR

The results of tRFs-seq were validated through qPCR, performed using the ViiA7 Real-time PCR System, rtStar™ tRF&tiRNA Pretreatment Kit (Arraystar, USA, #AS-FS-005), rtStar™ First-Strand cDNA Synthesis Kit (Arraystar, USA, # AS-FS-003), and 2×PCR master mix (Arraystar, USA, #AS-MR-005). The specific quantitative primers are listed in Table 2. The 10 μL reaction volume contained 0.5 μL of each primer, 2 μL of H_2_O, 2 μL of cDNA, and 5 μL of 2× Master Mix. The conditions were 95 °C for 10 min followed by 40 cycles (95 °C for 10 s and 60 °C for 60 s). Each experiment was performed in triplicate.

**Table 2:** Primers used in qPCR analysis.

**Accession No.**	**Primer sequence (5ʹ−3ʹ)**	**Product size**
AS-tDR-011775	F: TACAGTCCGACGATCGTGGT R: GTGCTCTTCCGATCTAAATTAACTA	49
AS-tDR-006835	F: CTACAGTCCGACGATCTCACG R: GTGCTCTTCCGATCTTGGTG	48
AS-tDR-013428	F: CTCCCTGGTGGTCTAGTGGTT R: TGTGCTCTTCCGATCTTATCCT	43
AS-tDR-010789	F: CTACAGTCCGACGATCCAGTC R: TGCTCTTCCGATCTAATGCTC	46
AS-tDR-005058	F: CTACAGTCCGACGATCCTTTG R: TGCTCTTCCGATCTACCCAC	46
AS-tDR-007919	F: ATCGTTTCCGTAGTGTAGTGGT R: CGTGTGCTCTTCCGATCTAAT	44
AS-tDR-008616	F: CCGACGATCTGGTAGAGCATT R: TGTGCTCTTCCGATCTACAGTCA	44
AS-tDR-009392	F: AGTCCGACGATCTGGTTAGG R: TGTGCTCTTCCGATCTAAGC	47
AS-tDR-010654	F: CTACAGTCCGACGATCTCCC R: TTCCGATCTAAATCCTAACCACTA	52
AS-tDR-011389	F: TCCGACGATCCCTGTCACGC R: TTCCGATCTGCCCCGGTCTC	42
AS-tDR-011670	F: GACGATCTGGTTAGGATTCGG R: TCTTCCGATCTAGCGGTGAG	45
AS-tDR-012690	F: AGTCCGACGATCTCCCCAG R: GCTCTTCCGATCTAGGTGGAG	43
AS-tDR-011438	F: AGTCCGACGATCTGCTTTGC R: TGTGCTCTTCCGATCTACCCA	46
U6	F: GCTTCGGCAGCACATATACTAAAAT R: CGCTTCACGAATTTGCGTGTCAT	89

### Target prediction

Research has shown that a highly important function of tRFs is to behave like miRNAs and repress the expression of endogenous targets [25, 26]. In other words, the tRFs pair with the 3′UTRs of the mRNAs to direct the latter’s post-transcriptional repression. Given this observation, we used miRanda and TargetScan to systematically predict tRF-mRNA interactions. In this research, the levels of the tRFs and mRNAs were different between the SAMP8 and SAMR1 mice and were thus further investigated. Our previous study [21] revealed that 482 mRNA transcripts were differentially expressed between SAMP8 and SAMR1 mouse brains at 7 months of age (adjusted *p* value < 0.05, Supplementary Table 5).

### Gene Ontology (GO) and Kyoto Encyclopedia of Genes and Genomes (KEGG) survey

GO enrichment analysis was applied to the target genes of tRFs. The GOseq R package was used to perform GO analysis [50]. GO terms with adjusted *p* value < 0.01 were recognized as significant enrichment. We used KOBAS software [51] to detect the enrichment of tRF target genes in KEGG pathways. Hypergeometric *P* value <0.01 was considered significant.

### Statistical analysis

The test results were analyzed by SPSS 20.0 and Graph pad prism 5 software. Box plot was conducted to show distribution of data into quartiles. The ends of the box were the upper and lower quartiles. The median was marked by a vertical line inside the box. The whiskers were the two lines outside the box that extended to the highest and lowest observations. Dot plot was used to show the qPCR data. *p* < 0.05 represents significant difference. The difference of the escape latency data in the MWM test was compared with two-way ANOVA. Student’s *t* test was used to compare the qPCR results and the remaining data of the MWM test.

## Supplementary Material

Supplementary Figures

Supplementary Tables

Supplementary Table 2

Supplementary Table 3

Supplementary Table 5
