# Supplementary material for: Identification of functional tRNA-derived fragments in senescence-accelerated mouse prone 8 brain
Source: Aging (Albany NY). 2019 Nov 20;11(22):10485–98. doi: 10.18632/aging.102471 (PMC6914438; doi:10.18632/aging.102471)
Supplement: Supplementary Figures [file aging-11-102471-s005..pdf]

SUPPLEMENTARY FIGURES

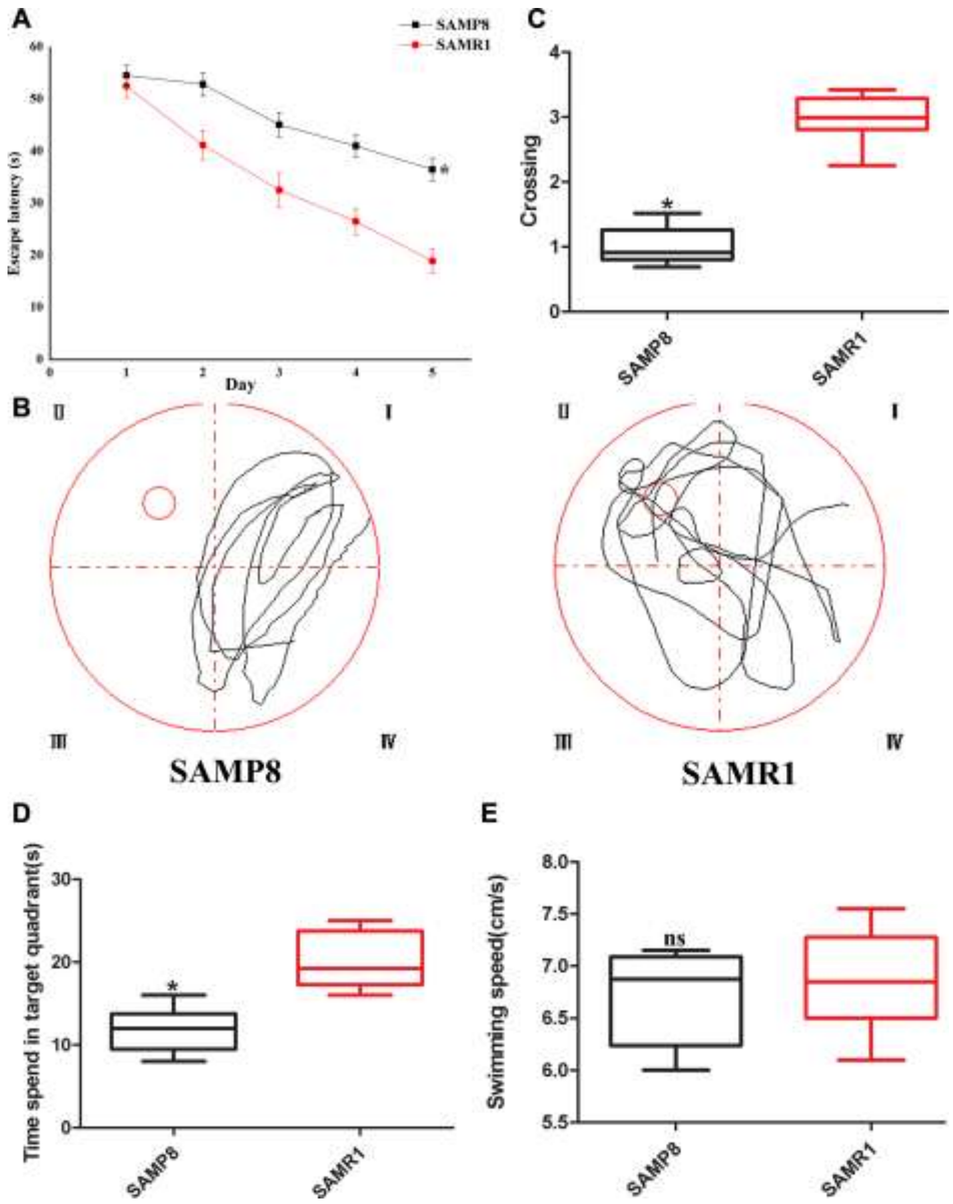

Supplementary Figure 1. Memory is impaired in SAMP8 mice. We used MWM to test to evaluate learning and memory in 7-month-old SAMP8 and SAMR1 mice (n=8/group). (**A**) Mean escape latency in the hidden platform experiment. (**B**) Swimming paths in the spatial probe experiment. (**C**) Number of crossings in the spatial probe experiment. (**D**) Time spent in the target quadrant in the spatial probe experiment. (**E**) Swimming speeds of the mice were similar between the two groups. *$p < 0.05$, ns means non-significant.

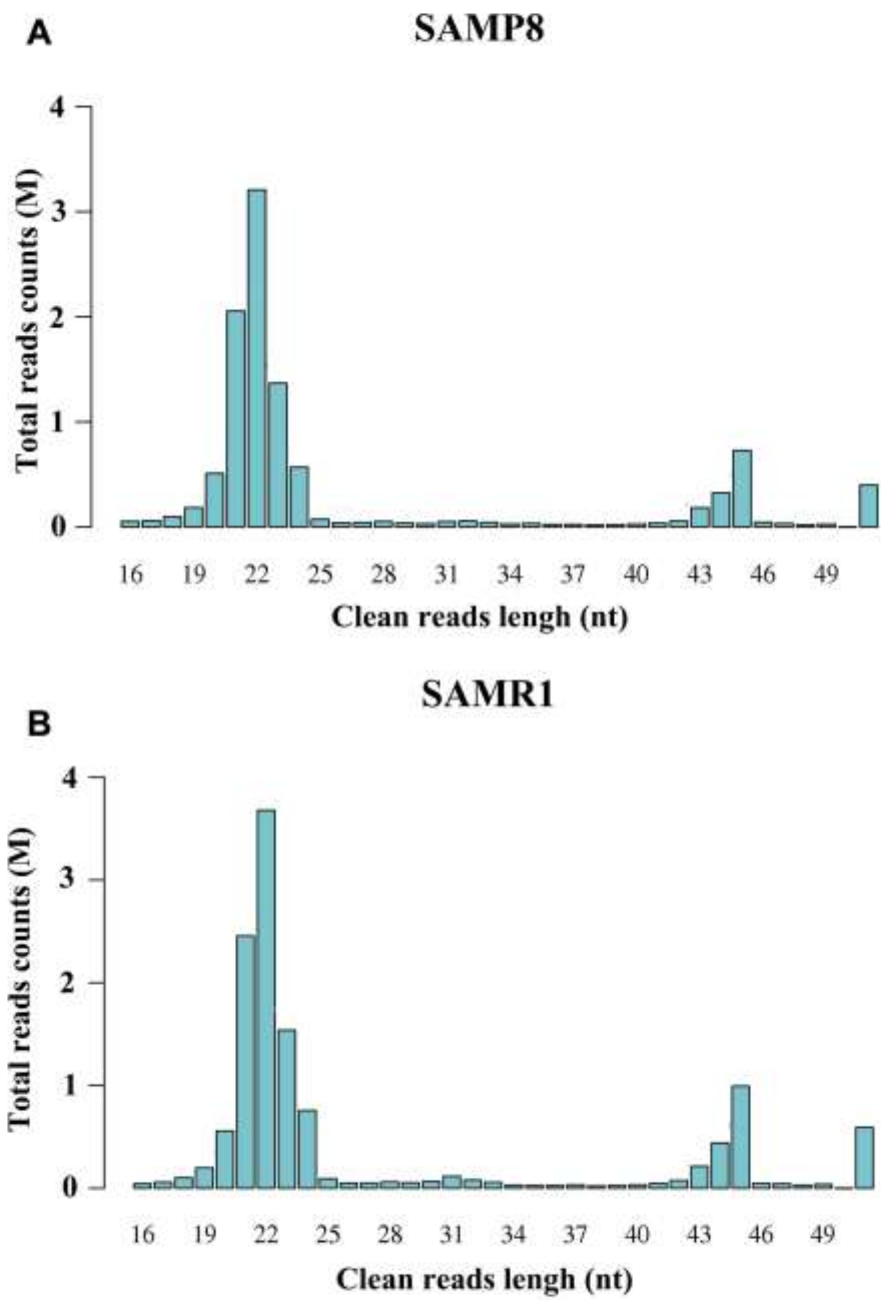

Supplementary Figure 2. Sequence length distribution of clean reads in the two groups. Sequence length distribution in (**A**) SAMP8 and (**B**) SAMR1 mice.
