# Supplementary material for: Identification of functional tRNA-derived fragments in senescence-accelerated mouse prone 8 brain
Source: Aging (Albany NY). 2019 Nov 20;11(22):10485–98. doi: 10.18632/aging.102471 (PMC6914438; doi:10.18632/aging.102471)
Supplement: Supplementary Tables [file aging-11-102471-s004..pdf]

SUPPLEMENTARY TABLES

Supplementary Table 1. Significantly and differentially expressed tRF transcripts between SAMP8 and SAMR1 mice.

tRFs_ID	source tRNA	Left	Right	tRF_Length	tRF_Sequence	SP8_TPM	SR1_TPM	fold_change	*p* value
AS-tDR-011775	pre-VAl-TAC-1-1	114	132	19	GTGGTGTGCTAGTTAATTT	162.75	0.00	163	5.6518E-05
AS-tDR-006835	Trp-CCA-1-1	59	75	17	TCACGTCGGGGTCACCA	179.04	0.00	179	0.00078074
AS-tDR-005058	Ser-GCT-3-1	48	63	16	CTTTGCACGCGTGGGT	204.88	0.00	205	0.00433379
AS-tDR-013428	Glu-CTC-2-1	1	26	26	TCCCTGGTGGTCTAGTGGTTAGGATA	0.00	192.05	-192	2.2536E-05
AS-tDR-010789	Lys-TTT-1-1	13	28	16	CAGTCGGTAGAGCATT	200.89	572.08	-2.84	0.00012432
AS-tDR-011389	Asp-GTC-2-1	31	53	23	CCTGTCACGCGGGAGACCGGGGC	0.00	176.24	-176	0.00103563
AS-tDR-012690	Ala-AGC-3-1	58	75	18	TCCCCAGCATCTCCACCT	0.00	196.14	-196	0.00281864
AS-tDR-011670	Glu-CTC-1-1	16	42	27	TGGTTAGGATTCGGCGCTCTCACCGCT	665.27	1731.64	-2.6	0.00428934

Please browse Full Text version to see the data of Supplementary Tables 2 and 3.

Supplementary Table 2. Potential targets of tRFs.

Supplementary Table 3. GO enrichment analysis of the tRF-targeting genes.

Supplementary Table 4. KEGG enrichment analysis of the tRF-targeting genes.

Term	Input number	Background number	*P* value
Protein processing in endoplasmic reticulum	5	167	0.0001361
Influenza A	5	172	0.0001556
Measles	4	139	0.0007561
Synaptic vesicle cycle	3	62	0.0008535
Axon guidance	4	176	0.0017676
Estrogen signaling pathway	3	98	0.0030246
Toxoplasmosis	3	113	0.0044611
Epstein-Barr virus infection	4	232	0.0046664
Prion diseases	2	34	0.0047169
Sphingolipid signaling pathway	3	125	0.0058631
Dopaminergic synapse	3	135	0.0072115
Spliceosome	3	135	0.0072115
Hepatitis C	3	137	0.0075014
Ribosome	3	148	0.0092182

Please browse Full Text version to see the data of Supplementary Table 5.

Supplementary Table 5. Significantly and differentially expressed mRNA transcripts between SAMP8 and SAMR1 mice.
